# Incidental finding of Zinner syndrome in a Greek military recruit: a case report of a rare clinical entity

**DOI:** 10.1186/s40779-019-0194-9

**Published:** 2019-02-14

**Authors:** Evangelos N. Symeonidis, Chrysovalantis Gkekas, Ioannis Tsifountoudis, Asterios Symeonidis, Christos Georgiadis, Vasileios Kalyvas, Apostolos Malioris, Michail Papathanasiou

**Affiliations:** 1 Department of Urology, 424 General Military Hospital of Thessaloniki, 56429 Thessaloniki, Greece; 2 Department of Radiology, 424 General Military Hospital of Thessaloniki, 56429 Thessaloniki, Greece

**Keywords:** Zinner syndrome, Seminal vesicle cyst, Renal agenesis, Greek, Military recruit, Youth

## Abstract

**Background:**

Zinner syndrome represents a rare congenital malformation of the urinary tract. It comprises a constellation of Wolffian duct anomalies and is almost exclusively encountered as a classic triad of seminal vesicle cysts, ejaculatory duct obstruction and renal agenesis. Patients can be either asymptomatic or symptomatic. Recently, minimally invasive surgical techniques have emerged, superseding traditional surgery for select symptomatic cases. Our case highlights the finding of a rare clinical syndrome that was incidentally detected during a routine mass screening of military recruits in the Greek Armed Forces.

**Case presentation:**

Herein, we present a case of a 19-year-old male who reported having a solitary right kidney when examined in a military training center of Northern Greece. No additional clinical information was available; thus, referral to a tertiary urology department for further investigation ensued. Imaging studies, namely, computed tomography and magnetic resonance imaging, revealed left renal aplasia, multiple left seminal vesicle cysts, and ejaculatory duct obstruction. Laboratory values and urinalysis were within normal range. Semen analysis was significant for cryptozoospermia. Our patient remained asymptomatic during the entire hospitalization. Long-term follow-up was recommended. Nevertheless, he declined further investigation and sought treatment in a private practice setting.

**Conclusions:**

This article aims to present the incidental diagnosis of a rare syndrome in a military setting. Population screening conducted in the armed forces permits the identification of undiagnosed diseases that warrant further investigation. To the best of our knowledge, this was the first report of Zinner syndrome in a military recruit and the second case cited of a Greek patient in the published literature. Regular follow-up is the key to timely intervention in conservatively managed cases.

## Background

Zinner syndrome, which was first described in 1914, comprises a rare congenital malformation of the genitourinary tract [1–4]. An aberrant development of the mesonephric duct and absence of the ureteric bud during embryogenesis leads to ipsilateral renal agenesis and atresia of the ejaculatory duct, which subsequently progresses to cystic dilation of seminal vesicles, a unique characteristic of this syndrome [3, 5, 6]. Patients can remain asymptomatic for an extended period [3–5]. Indeed, the clinical manifestation strongly correlates to the onset of sexual activity [6].

## Case presentation

A 19-year-old male was referred to our department after initial physical examination to a military training center as part of his compulsory enlistment for the Greek military service. During the examination, he reported having a solitary right kidney but was otherwise asymptomatic with no further relevant details. Neither a history of trauma nor previous surgeries were reported. Physical examination was unremarkable. He had no scars on the trunk and normal external genitalia. No pain provoked or masses felt during palpation. A digital rectal examination was not performed due to the patient’s preference. No sexual intercourse was reported at the time. Furthermore, no previous notable medical history for hereditary or acquired diseases was mentioned. The whole blood count and urinalysis results were within normal limits.

**Fig. 1:**
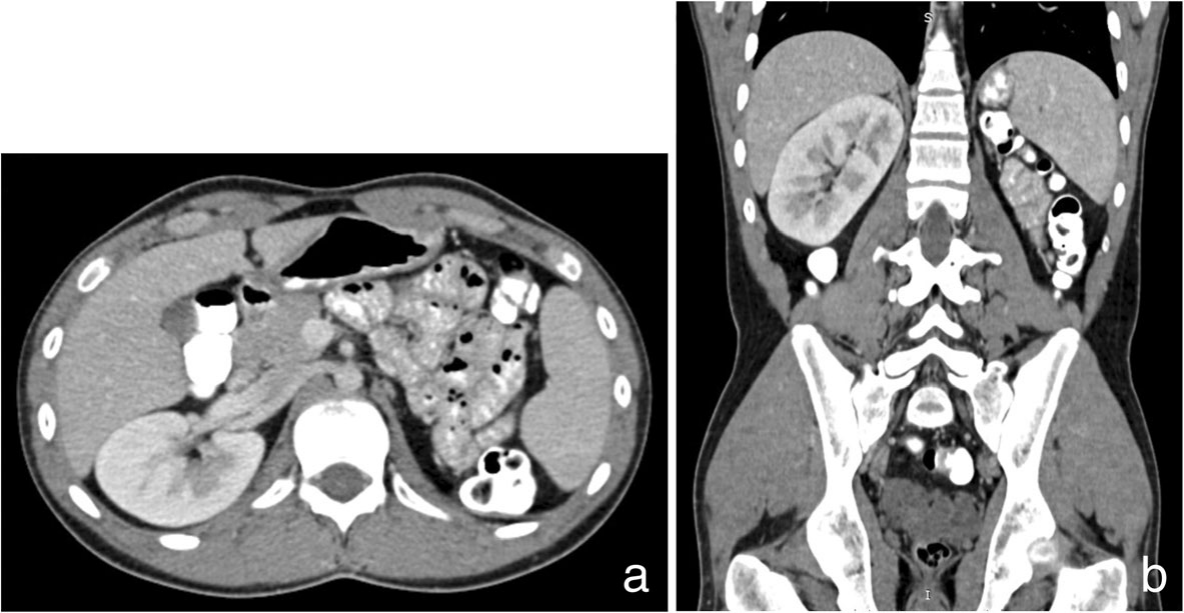
Contrast-enhanced axial (**a**) and coronal reconstruction (**b**) CT images of the abdomen reveal only a right kidney with absence of a left kidney

**Fig. 2:**
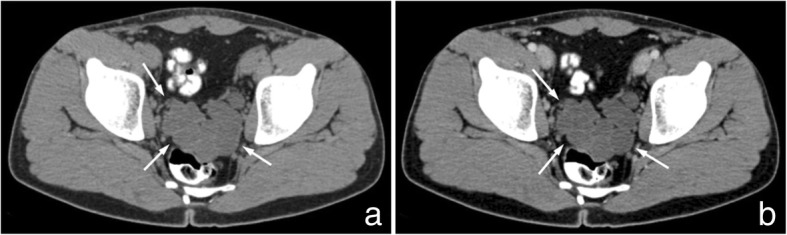
Axial CT image of the pelvis (**a**) depicts a large lobulated multicystic lesion of left seminal vesicle; the cystic lesion does not show enhancement on contrast-enhanced axial CT image (**b**) (arrows)

**Fig. 3:**
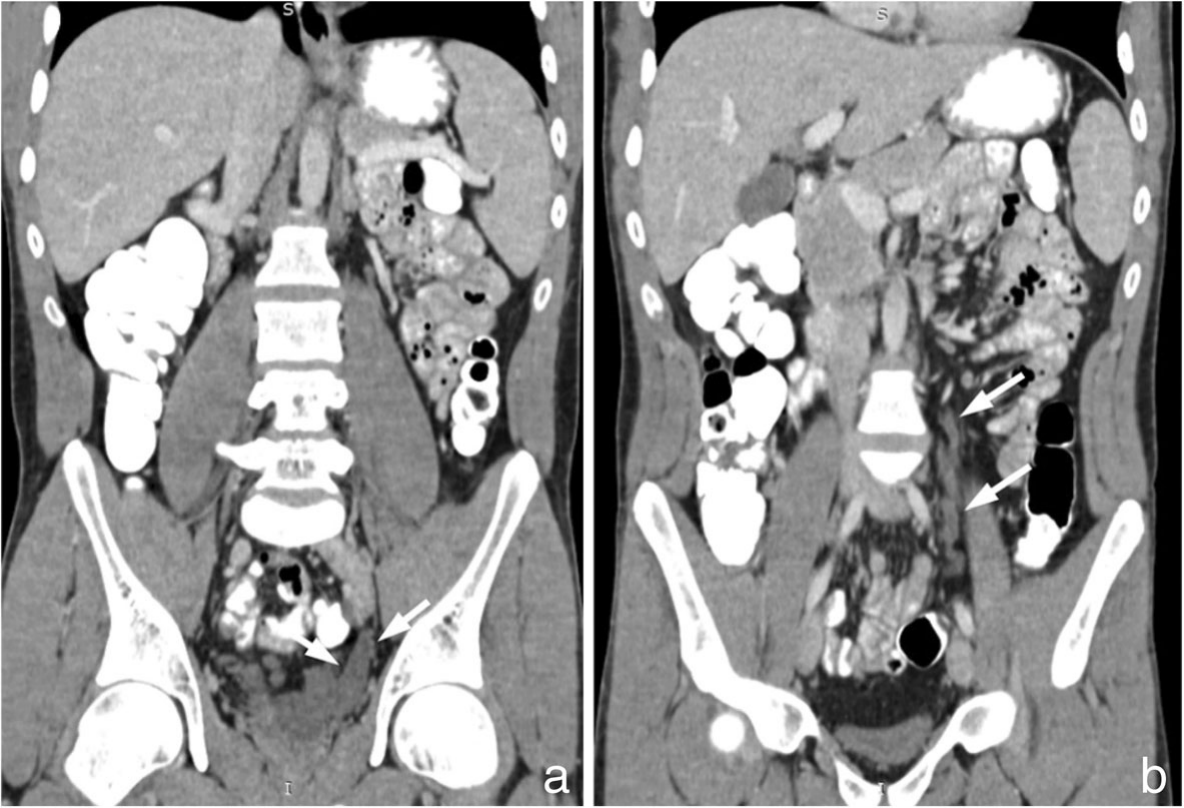
Contrast-enhanced coronal reconstruction CT images of the abdomen and pelvis depict a saccular dilated ectopic ureter opening into the left cystic seminal vesicle (**a**) and extending centrally up to the level of L_3_ vertebral body (**b**) (arrows)

Abdominopelvic computed tomography (CT) and magnetic resonance imaging (MRI) of the pelvis were performed. CT depicted only a right kidney with absence of left kidney (Fig. 1). Additionally, CT demonstrated a large lobulated multicystic lesion of left seminal vesicle without enhancement on contrast-enhanced images (Fig. 2). A saccular dilated ectopic ureter opening into the left cystic seminal vesicle and extending centrally up to the level of L_3_ vertebral body was revealed with a length of approximately 16 cm (Fig. 3).

MRI was performed with a Siemens Magnetom Avanto (1.5 Tesla) MRI unit (Siemens Inc., Germany) using a pelvic phased-array coil. The imaging protocol comprised T_1_-weighted, T_2_-weighted, T_2_-weighted with fat saturation (FS) and T_1_-weighted FS images on axial, sagittal and coronal planes. Finally, images on T_1_-weighted FS sequence after intravenous administration of contrast medium (gadolinium) were added.

**Fig. 4:**
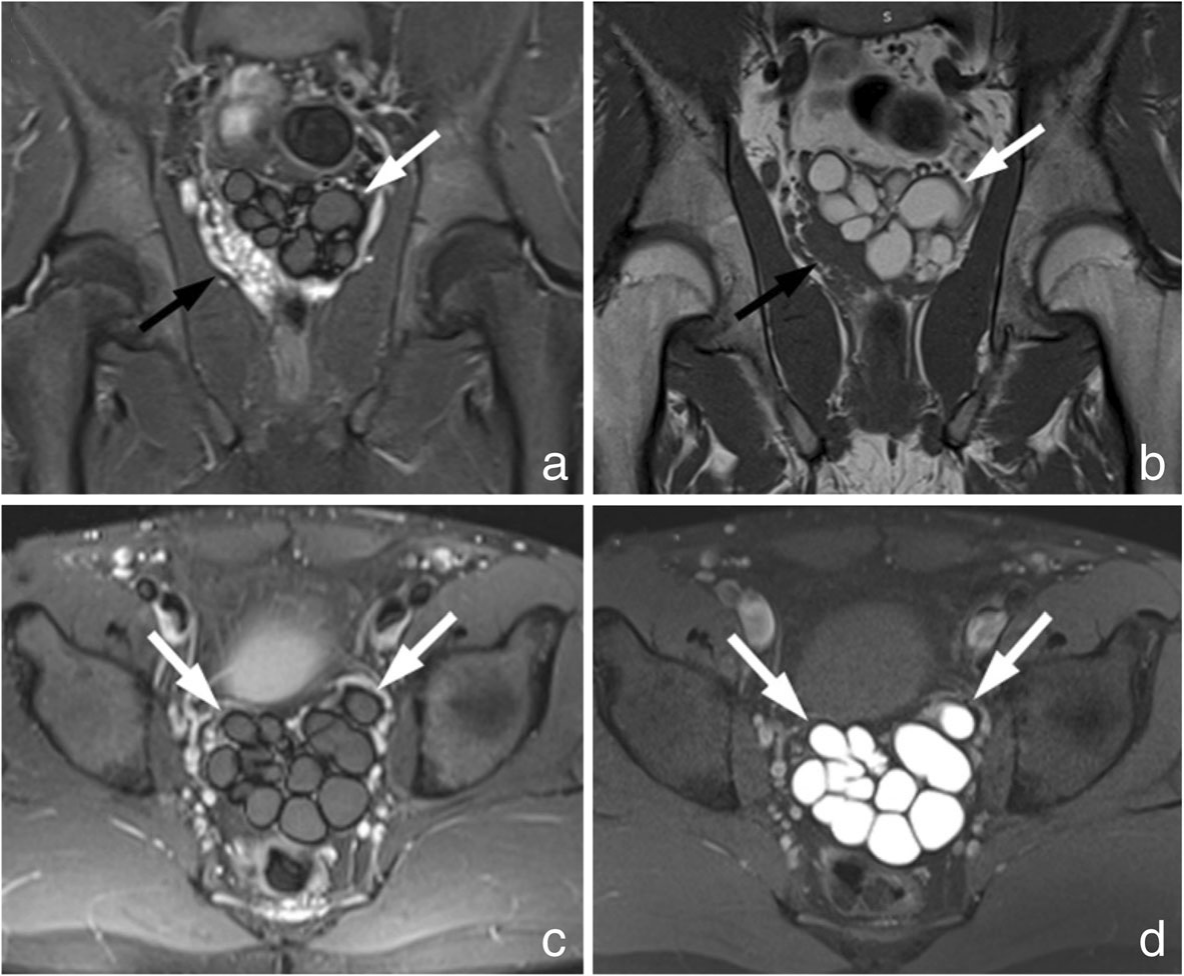
Coronal MR images demonstrate a large lobulated multicystic lesion in the anatomic region of left seminal vesicle with low signal intensity on T_2_-weighted FS images (**a**) and high signal on T_1_-weighted images (**b**), corresponding to a dilated SVC (white arrows). The multilobulated SVC is also depicted on axial T_2_-weighted FS (**c**) and T_1_-weighted FS (**d**) images with low signal and high signal intensity, respectively, suggesting proteinaceous or hematic content (white arrows). The normal right seminal vesicle is shown on coronal T_2_-weighted FS (a) and T_1_-weighted (**b**) images with high and intermediate signal intensity, respectively, corresponding to fluid (black arrows)

**Fig. 5:**
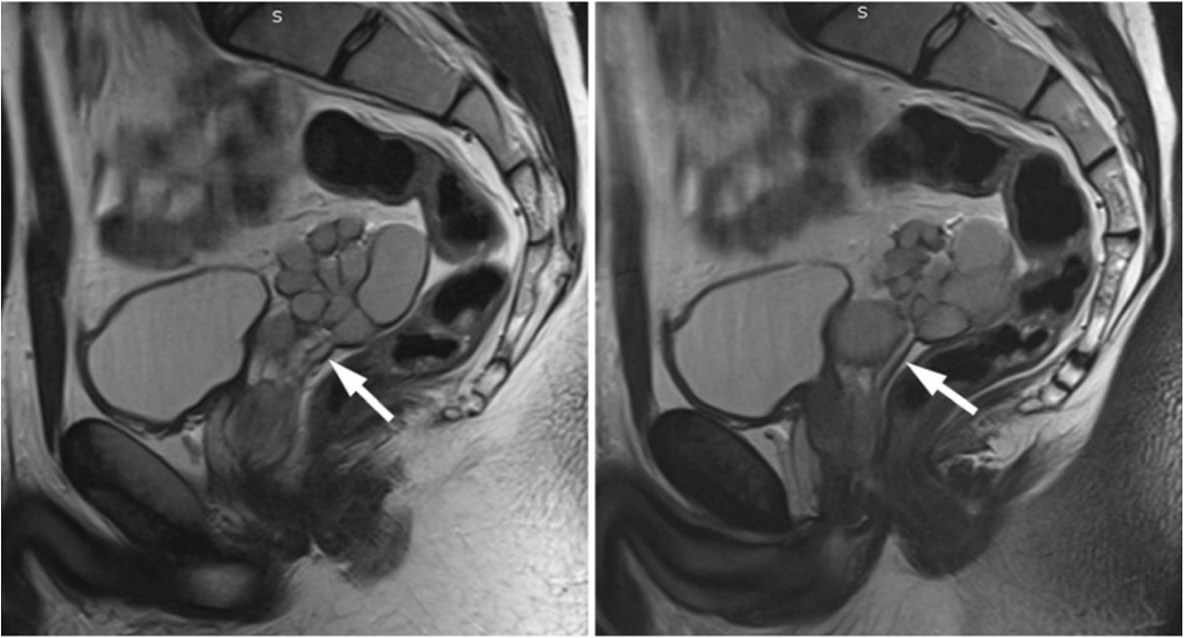
Sagittal T_2_-weighted MR images of the pelvis demonstrate enlargement of left ejaculatory duct communicating with the dilated SVC (arrows)

**Fig. 6:**
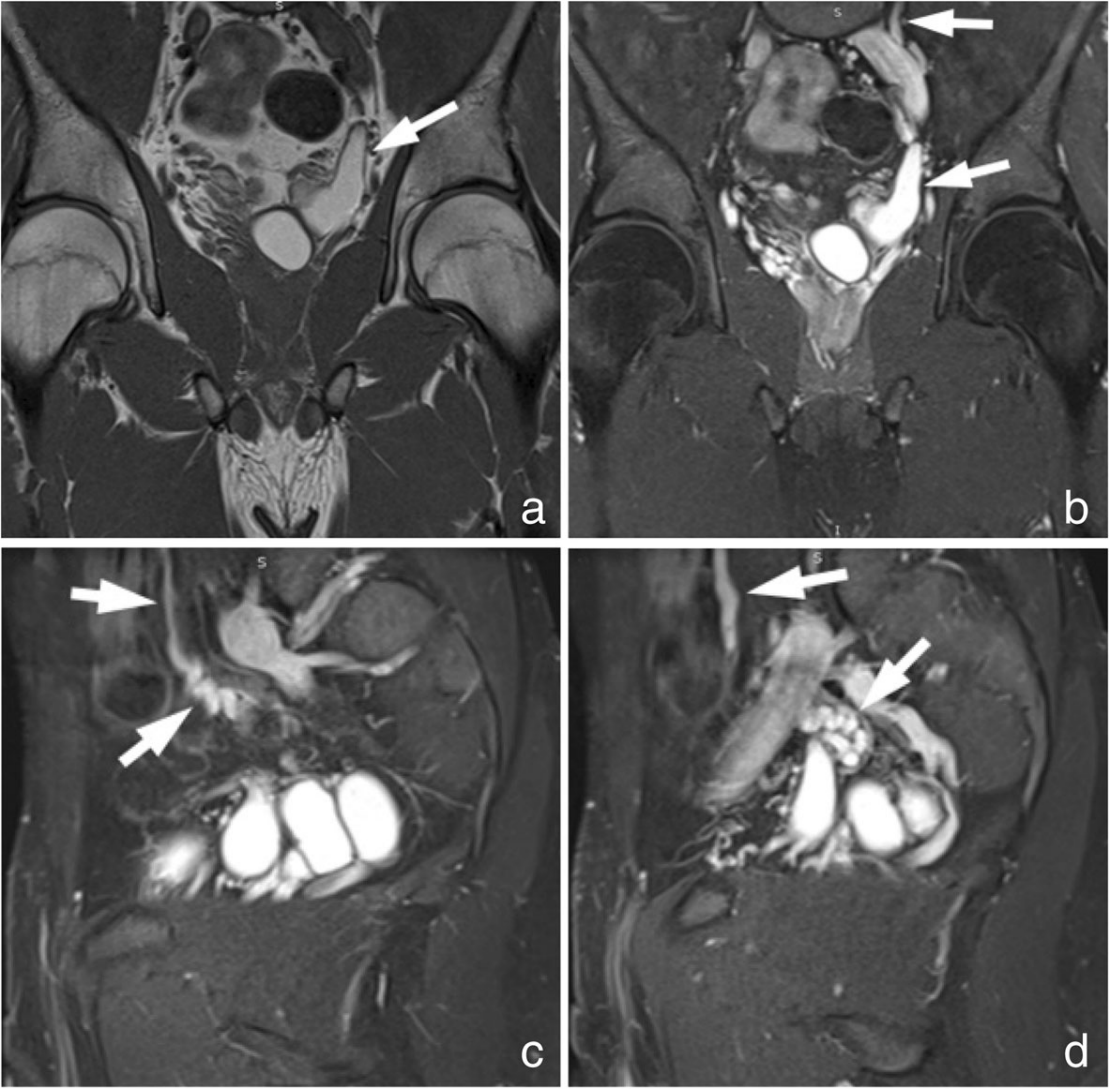
Coronal T_1_-weighted (**a**) and corresponding T_1_-weighted FS image after intravenous administration of contrast medium (**b**) depict a saccular dilated ectopic left ureter opening into the multilobulated SVC. The ectopic ureter is also observed extending centrally with a tortuous morphology on adjacent T_1_-weighted FS images after intravenous administration of contrast medium (**c** and **d**). High signal intensity in the ureter indicative of proteinaceous or hematic content is present on all MR images (arrows)

MRI demonstrated a large lobulated multicystic lesion in the anatomic region of left seminal vesicle. The lesion measured approximately 7.2 cm × 6.1 cm with low signal intensity on T_2_-weighted FS and high signal on T_1_-weighted images, corresponding to a dilated seminal vesicle cyst (SVC). The high signal intensity on T_1_-weighted sequences was strongly suggestive of proteinaceous or hematic content. In contrast, the normal right seminal vesicle exhibits high and intermediate signal intensity on T_2_-weighted FS and T_1_-weighted images, respectively, corresponding to fluid (Fig. 4). An enlargement of the left ejaculatory duct communicating with the dilated SVC was well depicted on sagittal T_2_-weighted images (Fig. 5). A saccular dilated ectopic left ureter with tortuous morphology, which was also filled with proteinaceous or hematic content, was revealed on all T_1_-weighted images, communicating with the SVC and extending centrally (Fig. 6).

Unfortunately, the patient declined additional investigation by means of transrectal ultrasonography (TRUS), which might have assisted in clarifying the ejaculatory duct obstruction.

Semen analysis revealed cryptozoospermia (volume < 1 ml, pH 8.0, total sperm count 126 /ml) of obstructive origin. Nevertheless, fertility was not the patient’s primary concern. He declined further management, although he was made aware of the rarity of the syndrome and the possible future need of surgical management. Despite detailed analysis of the importance of cryopreservation and fertility maintenance, he had no intention to cryopreserve sperm or undergo microsurgical sperm retrieval at the time. Therefore, annual semen analysis was recommended for as long as he presented with altered fertility status. Although SVCs are generally benign and rarely symptomatic, cases of malignant transformation and late diagnosis due to the absence of warning symptoms have been described [7]. Thus, we adequately informed our patient about the possibility of the carcinomatous evolution of SVCs. Finally, we advised him to undergo ultrasonographic monitoring on an annual basis.

## Discussion

Zinner syndrome is characterized by the triad of renal agenesis, ipsilateral SVC and ejaculatory duct obstruction [2, 8]. It is considered as the male counterpart of Mayer-Rokitansky-Kustner-Hauser female syndrome [3, 9].

The majority of patients typically remain asymptomatic until the second or third decade of life [2, 6]. Nevertheless, older patients have been equally affected and been treated accordingly [2, 5]. Clinical manifestations typically reach a peak during the period of increased sexual activity. Hematospermia, recurrent episodes of visible hematuria and ejaculation failure are the most commonly associated symptoms [1, 6]. Nonspecific symptoms, namely intermittent scrotal pain, perianal discomfort, and urinary frequency, have also been reported [3, 6, 9].

In our case, it is important to emphasize the incidental and prompt diagnosis of the syndrome in an otherwise asymptomatic young adult during enlistment screening. The initial screening, which was performed from the onset of the enlistment process, ensures health promotion and enhances the soldier’s future capacity for the remaining period of compulsory military service. The Greek armed forces implement an annual screening protocol for permanent staff, i.e., commissioned officers and enlisted soldiers, allowing for the identification of undiagnosed diseases that necessitate further investigation.

Although the syndrome was diagnosed in the context of the regular enlistment process, the investigation employed was neither typical of a mass screening nor could it be adopted as such. Our patient was investigated for a reported solitary kidney, which was subsequently proven to be left renal agenesis. Every step of the detailed imaging work-up was indispensable with regard to the fixed setting of the armed forces in which every disease reflects on the patient’s physical activity status. To the best of our knowledge, this is the first case of Zinner syndrome described in a military setting and the second case with a Greek patient in the current body of literature since the first report by Kyriakidis et al. [10] in 1995.

Our diagnostic approach was based on physical examination, abdominopelvic CT and pelvic MRI. Both CT and MRI are reliable diagnostic modalities [1, 5]. The utility of CT imaging is manifold; namely, it can reliably distinguish an ectopic kidney that could be easily missed for agenesis. It can also provide an initial assessment of the pelvic cavity for associated congenital anomalies. Although CT may reveal kidney agenesis and SVC as well as the remnant ureter when present, it may be inadequate to establish the diagnosis [11]. Given multiplanar imaging and the use of different sequences, MRI can accurately evaluate all pelvic organs and adjacent soft tissue structures. It may also precisely characterize any pelvic mass using sequences after intravenous administration of contrast medium [5, 8]. In this manner, it can contribute to surgical treatment planning in addition to establishing a definitive diagnosis [5, 11].

On MRI, SVCs may appear with variable signal intensity. Their content exhibits signal intensity generally analogous to fluid, i.e., high signal on T_2_-weighted and intermediate on T_1_-weighted sequences, without enhancement after intravenous gadolinium administration. However, increased T_1_-weighted and decreased T_2_-weighted signal intensity may also be encountered, which is attributable to hemorrhage or increased concentration of proteinaceous fluid, as noted with our case [12]. Occasionally, MRI can depict the communication between SVC and ectopic dilated ureter, which may also contain hemorrhagic or proteinaceous fluid, as in our case [13]. In selected cases, such as ours, enlargement of an obstructed ejaculatory duct communicating with the dilated SVC may be demonstrated [11].

The differential diagnosis includes a variety of entities from prostate and ejaculatory duct cysts to bladder diverticula and ureterocele [1, 5]. Contrary to our case, van den Ouden et al. [6] demonstrated a right-sided preponderance of the syndrome. In 2015, Kanavaki et al. [3] reported the case of a 4-year-old boy who presented with a right paravesical cyst on ultrasound that was initially diagnosed as ureterocele. Eventually, after 11 years of annual ultrasonographic follow-up, the diagnosis of Zinner syndrome was established. Interestingly, during the same year, Pavan et al. [14] cited a unique case of the syndrome in a middle-aged male with a palpable, painless paratesticular mass mimicking a varicocele.

Surgery is the gold standard approach for symptomatic cases [1, 4–6]. Currently, minimally invasive techniques have outpaced traditional open surgery [1, 2, 8, 15, 16]. Recently, Kord et al. [15] described one of the most extensive series of 5 patients treated with minimally invasive surgery (MIS). They found that MIS was feasible and efficient, providing many advantages for both the patient and surgeon. In 2018, Kiremit et al. [17] reported the successful robotic repair of the syndrome in a 23-year-old male with a 2-year history of lower urinary tract symptoms, perineal pain, and recurrent urinary tract infections. Previously, various robotic-assisted approaches have been presented with favorable outcomes [2, 16, 18]. Fertility status remains of considerable concern and should be thoroughly investigated. Even in surgically treated cases, infertility might persist, and assisted reproduction techniques appear as the only alternative [1].

## Conclusion

In conclusion, screening the population employed in the Greek Armed Forces may lead to the early diagnosis of rare diseases. Urologists should consider adding Zinner syndrome in their differential diagnosis when they encounter a solitary kidney in an otherwise asymptomatic patient. A thorough radiologic investigation of the abdomen and pelvis, including CT and MRI, is warranted in the presence of kidney anomalies. Moreover, radiologists should be aware of this rare entity and maintain a high index of suspicion when encountering analogous imaging findings. The need for long-term follow-up in patients presenting with this pathology assures prompt surgical intervention when clinical symptoms emerge or fertility is affected.

## Abbreviations

CT: Computed tomography
FS: Fat saturation
MIS: Minimally invasive surgery
MRI: Magnetic resonance imaging
SVC: Seminal vesicle cyst
TRUS: Transrectal ultrasonography
